# Sequencing Methods to Study the Microbiome with Antibiotic Resistance Genes in Patients with Pulmonary Infections

**DOI:** 10.4014/jmb.2402.02004

**Published:** 2024-06-20

**Authors:** Tingyan Dong, Yongsi Wang, Chunxia Qi, Wentao Fan, Junting Xie, Haitao Chen, Hao Zhou, Xiaodong Han

**Affiliations:** 1 Integrated Diagnostic Centre for Infectious Diseases, Guangzhou Huayin Medical Laboratory Center, Guangzhou, P.R. China; 2 Immunology and Reproduction Biology Laboratory & State Key Laboratory of Analytical Chemistry for Life Sciences, Medical School, Nanjing University, Nanjing, P.R. China; 3 Department of Hospital Infection Management, NanFang Hospital, Southern Medical University, Guangzhou, P.R. China; 4 Jiangsu Key Laboratory of Molecular Medicine, Nanjing University, Nanjing, P.R. China

**Keywords:** Pulmonary infections, metagenomic next-generation sequencing, reads-based and assemblybased method, antibiotic-resistant bacteria, antibiotic resistance genes

## Abstract

Various antibiotic-resistant bacteria (ARB) are known to induce repeated pulmonary infections and increase morbidity and mortality. A thorough knowledge of antibiotic resistance is imperative for clinical practice to treat resistant pulmonary infections. In this study, we used a reads-based method and an assembly-based method according to the metagenomic next-generation sequencing (mNGS) data to reveal the spectra of ARB and corresponding antibiotic resistance genes (ARGs) in samples from patients with pulmonary infections. A total of 151 clinical samples from 144 patients with pulmonary infections were collected for retrospective analysis. The ARB and ARGs detection performance was compared by the reads-based method and assembly-based method with the culture method and antibiotic susceptibility testing (AST), respectively. In addition, ARGs and the attribution relationship of common ARB were analyzed by the two methods. The comparison results showed that the assembly-based method could assist in determining pathogens detected by the reads-based method as true ARB and improve the predictive capabilities (46% > 13%). ARG-ARB network analysis revealed that assembly-based method could promote determining clear ARG-bacteria attribution and 101 ARGs were detected both in two methods. 25 ARB were obtained by both methods, of which the most predominant ARB and its ARGs in the samples of pulmonary infections were *Acinetobacter baumannii* (*ade*), *Pseudomonas aeruginosa* (*mex*), *Klebsiella pneumoniae* (*emr*), and *Stenotrophomonas maltophilia* (*sme*). Collectively, our findings demonstrated that the assembly-based method could be a supplement to the reads-based method and uncovered pulmonary infection-associated ARB and ARGs as potential antibiotic treatment targets.

## Introduction

With the emergence of new pathogens and an increasing number of antibiotic-resistant bacteria (ARB), the incidence and mortality rate of pulmonary infection patients continue to increase [1, 2]. It is imperative for clinical practice to identify and understand antimicrobial resistance to treat resistant infections [3]. In recent years, advancements in metagenomic next-generation sequencing (mNGS) have expanded our abilities to detect and study ARB and the corresponding antibiotic resistance genes (ARGs)[4]. However, the diagnostic criteria of mNGS are not uniform, and it is not clear which approaches are the most accurate for interpreting mNGS results, including the judgment of false-positive predictions [5]. In this study, reads-based methods and assembly-based methods according to mNGS data for the comprehensive detection of ARB and ARGs were explored to improve diagnostic results.

For the reads-based method, ARGs in a sample can be detected by mapping reads to the reference databases using alignment tools such as Bowtie 2 [6] or BWA (Burrows‒Wheeler-Alignment) [7] or by splitting reads into k-mers and mapping them to the reference databases [5]. Reads-based methods enable the identification of ARGs from low-abundance organisms present in complex communities. Therefore, it has gained traction in recent years, especially in clinical diagnostics where conducting real-time sequencing-based resistance prediction is crucial [8]. However, mapping reads directly to large datasets was likely to result in false-positive predictions, as reads derived from protein coding sequences may spuriously align to other genes as a result of local sequence homology [5, 9]. Meanwhile, reads-based methods depend largely on curated antibiotic resistance databases that are comprehensive and contain all variants of the reference gene information [10]. Regarding the assembly-based method, sequencing reads are first assembled into contiguous fragments (contigs) using several metagenome-specific assemblers, such as IDBA-UD [11], MEGAHIT [12] or MetaSPAdes [13], and then annotated by comparison with custom or public reference databases to detect ARGs [5]. The assembly-based method, according to the contig facilitates, can identify ARGs that are more divergent from and lack homology to known sequences in the reference databases [5]. However, the process of de novo assembly and annotation is computationally expensive, time consuming and requires higher genome coverage [5]. Both methods have trade-offs. Presently, there is no consensus on which data analysis method is better, and the choice of analysis mainly depends on the type of sequencing, availability of computational resources and the study objective [5, 14, 15]. Given the above, we explored whether a combination of assembly-based and reads-based methods could be better than a single method using our in-house sequencing data to improve the performance of antibiotic resistance diagnostic results.

In this study, first, ARG and ARB detection rates were analyzed by the reads-based method and assembly-based method according to the methods introduced in the literature. Then, the consistency between the ARB and clinical practice was compared by the two methods with culture testing and AST results. We further performed ARG-ARB network analysis to provide bioinformatics-oriented evidence for the detection results. Finally, the common results obtained by the two methods were comprehensively evaluated as the main ARB and their corresponding ARGs in patients with pulmonary infection. Our study demonstrates that the comprehensive application of two methods better assesses antibiotic resistance than conventional methods and single methods. Collectively, this study provided a profile of the predominant ARB and ARGs from pulmonary infection patients by comparing the reads-based method and assembly-based method to improve clinical antibiotic resistance diagnostic results and potentially better treat these infections.

## Materials and Methods

### Participants and Sample Collections

In this retrospective study, 151 clinical specimens from 144 patients with pulmonary infections, including bronchoalveolar lavage fluid (BALF) and sputum samples, were collected for clinical mNGS testing at Huayin Medical Center (China) from January 2022 to March 2023. All patients were diagnosed with pulmonary infections judged by a board-certified physician on the basis of diagnostic criteria, clinical manifestations and imaging examination results. Basic information, including age, sex, department, and comorbidities, was extracted from their medical records. The clinical laboratory results for microbiological culture and antimicrobial susceptibility testing (AST) were also extracted for analysis. The bacterial culture was streaked onto 10 μl of samples on blood agar and MacConkey agar plates and then incubated under aerobic conditions at 37°C for 48 h. The microorganisms were identified using the VITEK 2 automated system. Accordingly, if no microorganisms were isolated, samples were reported as culture negative. AST was performed following the guidelines of the Clinical and Laboratory Standards Institute (CLSI)[16]. This study was approved by the Ethics Committee of Huayin Medical Laboratory Center (2022-004-02). The participants provided their written informed consent to participate in this study.

### Library Preparation and mNGS Sequencing

**Fig. 1:**
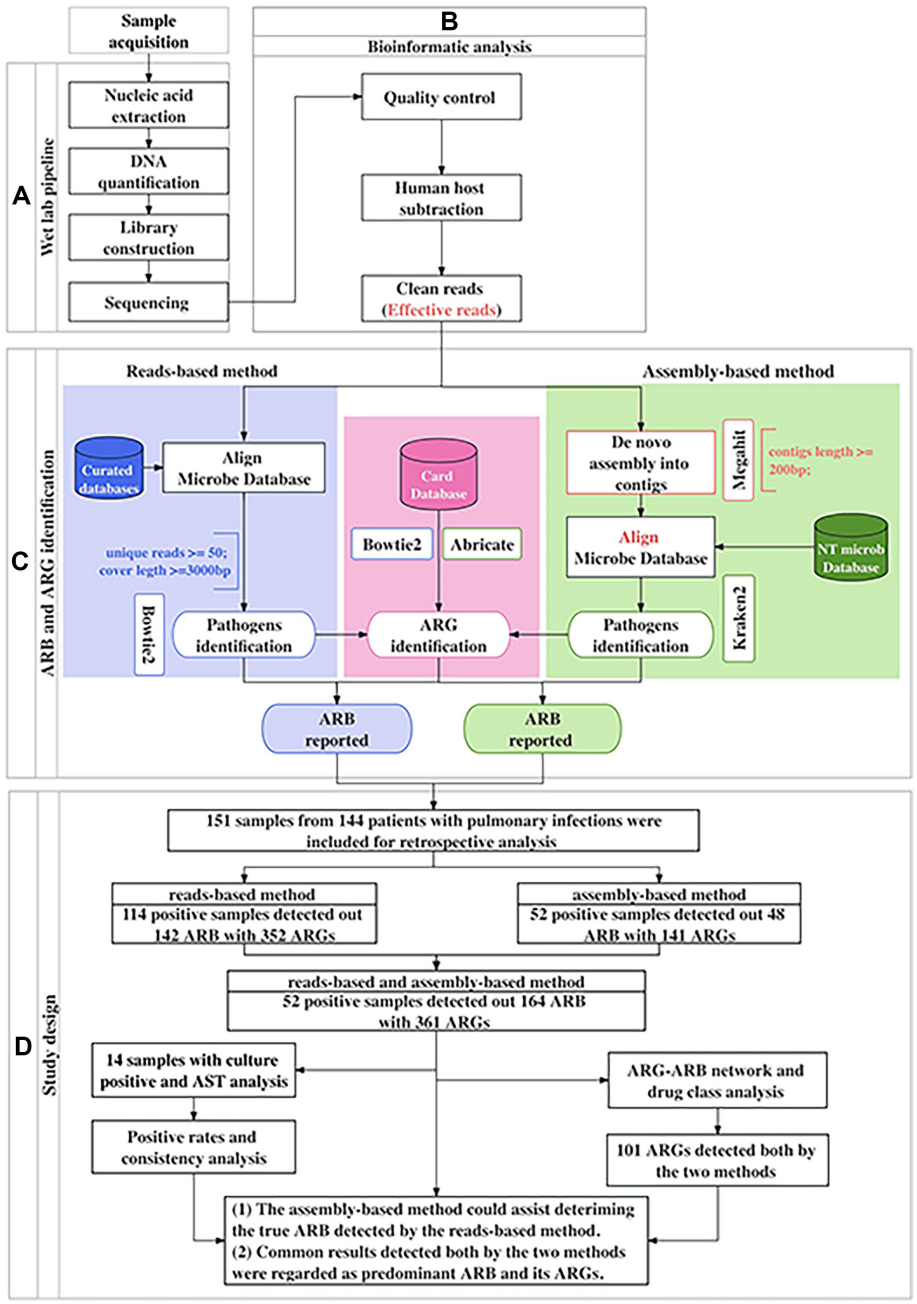
Process of method analysis and study design. (**A**) Flowchart of analysis by web experiments. (**B**) Bioinformatics analysis for mNGS data. (**C**) ARB and ARGs identification pipelines with the reads-based method and assembly-based method. (**D**) Study design workflow.

Clinical samples were collected from each patient by following aseptic processing procedures [17]. The detailed process is described in Fig. 1A and 1B. (1) Nucleic acid extraction and quantification Bronchoalveolar lavage fluid (BALF) at least 5 ml and 1–3 ml from sputum were subjected to DNA extraction using a TIANamp Micro DNA Kit (DP316, Tiangen Biotech Co., China). (2) Library construction A DNA library was constructed with the VAHTS Universal Plus DNA Library Prep Kit for Illumina (ND617-C2, Vazyme Biotech Co., China) according to the manufacturer’s protocol. (3) Sequencing An Agilent 2100 Bioanalyzer was used to analyze the length of the inserted fragments in the library. A Qubit dsDNA HS assay kit was used to control the concentration of the library. Qualified libraries were sequenced by the Illumina NextSeq 550 platform. The sequencing model was SE50 (Single-end 50 bp) and the amount of single-end sequencing data was about 5 million reads count. (4) Data Filtering High-quality sequencing data (clean reads) were generated by removing the low-quality reads and short reads (length < 50 bp) by Fastp software (v.0.20.1). To ensure effective microbial data (effective reads), the reads were compared to the version of grch38.p12 of the human genome using Kraken 2 and BWA software to filter out residual human source read pollution that could not be removed by experimental means, and only the microbial reads were reserved for subsequent classification. The effective reads were used to analyze pathogens or antibiotic resistance genes by reads-based methods or assembly-based methods. The basic read statistics for sequencing data and assembly statistics for each sample were showed in the Table S1. The classification reference databases were downloaded from the National Center for Biotechnology Information (NCBI) (ftp://ftp.ncbi.nlm.nih.gov/genomes/).

### Identification of Positive ARB and ARGs

**Reads-Based Method.** Antibiotic resistance genes in a sample can be detected by aligning reads to the reference databases using the alignment tool Bowtie 2 [6].

Threshold criteria for bacteria and ARGs as a positive result in the reads-based method:

(i) For bacteria: bacteria species whose filtered data were aligned to the Pathogenic Microbial Genome Databases by Bowtie 2, including contained 1,410 bacterial species, 1,312 viral species, 88 fungal species, 56 parasites (Table S2). The filter conditions were of the first rank with unique reads ≥ 50 and cover length ≥ 3000 bp.

(ii) For ARGs, the filtered data were aligned to the Comprehensive Antibiotic Resistance Database (CARD) by Bowtie 2. Classification of ARG resistance mechanisms was carried out according to the CARD (CARD Prevalence, Resistomes, & Variants data 3.1.0) database. The filter conditions used were as follows: unique reads ≥1, unique ratio (Uratio) ≥10%, unique read coverage ratio ≥ 30%, and unique read coverage index (Uindex) > 30%.

$$\text{Uratio = }\left(\frac{\text{uN}}{\text{aN}}\right)\text{*100\%}$$

Uratio: unique read ratio (how many aligned reads are unique reads in X species)

*uN: number of unique reads mapped to X species.*

*aN: number of all reads mapped to X species.*

$$\text{Uindex = }\left(\frac{\text{sizeR}}{\text{sizeC}}\right)\text{*100\%}$$

*sizeR: real coverage of unique reads.*

sizeC: coverage of unique reads (when calculated coverage < genome size: sizeC = number of unique reads * 50, then coverage ≥ genome size: sizeC = genome size).

(iii) For ARB, when a ARG is detected and, simultaneously, the related bacteria are also identified in the sample (based on the CARD database), the bacteria associated with the ARG are considered ARB in that particular sample.

**Assembly-Based Method.** Sequencing reads were first assembled into contiguous fragments (contigs) by the metagenome-specific assembler MEGAHIT[12] and then annotated by comparison with CARD to detect ARGs in this study.

Threshold criteria for bacteria and ARGs as a positive result in the assembly-based method:

(i) For bacteria: the filtered data were assembled by MEGAHIT. The filtering conditions for contiguous fragments (contigs) were length ≥ 200 bp and min-count ≥ 2. Based on the contig sequences, species annotation was performed by Kraken 2 [18]. We retained the results only of contig species that could be annotated to the species level using Kraken 2, which specified sequence homology.

(ii) For ARGs: ARGs detected in contigs were processed by Abricate 0.8 software (http://github.com/tseemann/abricate) and were aligned to the CARD. The filter condition was identity ≥ 75% and coverage ≥ 50% for gene annotations.

(iii) For ARB: Bacteria with those contigs were defined as positive ARB in that sample if the contigs were annotated with bacteria and ARG information.

### Study Design

151 samples were collected for the antibiotic resistance analysis by the reads-based method and assembly-based method. To compare the ARB identification performance by the reads-based method and assembly-based method and making the results more accurate, 52 both positive samples by the two methods were used for comprehensive evaluation. Then, 14 culture-positive samples were subjected to consistency ratio analysis combined with reads-based method and assembly-based method analysis. Finally, the predominant ARB and its ARGs in patients with pulmonary infection were assessed based on the common results obtained by the two methods (Fig. 1D, Table S3).

### Definitions

Unique reads: the number of unique reads of standardized species

Effective reads: The remaining data that filtered out the residual human source read pollution.

Coverage: percentage of the length of the nucleic acid sequence detected over the total length of the genome of the microbe

ARG-bacteria attribution: an unknown ARG assigned to a particular bacterium.

### Statistical Analysis

SPSS Statistics 21.0 was used for clinical data analysis. Continuous measurement data that followed a normal distribution are shown as the means ± standard deviations (x ± s), and the Pearson chi-squared (χ^2^) test or Fisher’s exac*t* test was used for the comparison of frequencies of categorical data. Data with a nonnormal distribution are shown as the medians (interquartile ranges). A *P* value of 0.05 was considered statistically significant. The analysis and visualization workflow of the bar graph and heatmap diagram used the ggplot2 R package to estimate the ARB detection by the two methods. The Sankey diagram was used to visualize the ARG-ARB attribution relationship and was conducted in R using the “Sankey Network” function in the R package “networkD3.” The Venn diagram was used to visualize using the gplots R package. The Upset diagram used the Upset R package to visualize the distribution of ARB in different groups.

## Results

### Patient and Sample Characteristics

In this study, the clinical information of 144 patients with pulmonary infections was retrospectively analyzed. The mean age of all patients was 67.1 years, ranging from 15 to 95. Among them, 77.0% of patients were male. A total of 61.1% of patients had underlying comorbidities, including respiratory failure (32, 22.2%), disorders of consciousness (14, 9.7%), cardiovascular disease (9, 6.3%), malignancies (11, 7.6%), and diabetes (6, 4.2%). The ARB-positive results were associated with the department (*P* = 0.001) but not gender, age, antibiotic history or comorbidities (Table 1). Primary mNGS analysis was performed for 151 samples (134 BALF, 17 sputum ), of which 114 positive samples were detected by the reads-based method, and 52 positive samples were detected by the assembly-based method (Table S3). The ARG-positive rates for the reads-based method were two time higher than the assembly-based method (75.5%, 114/151 vs. 34.4%, 52/151, *P* < 0.05).

### Comparison of the Reads-Based Method and Assembly-Based Method with the Conventional Method for the Identification of ARB

**Fig. 2:**
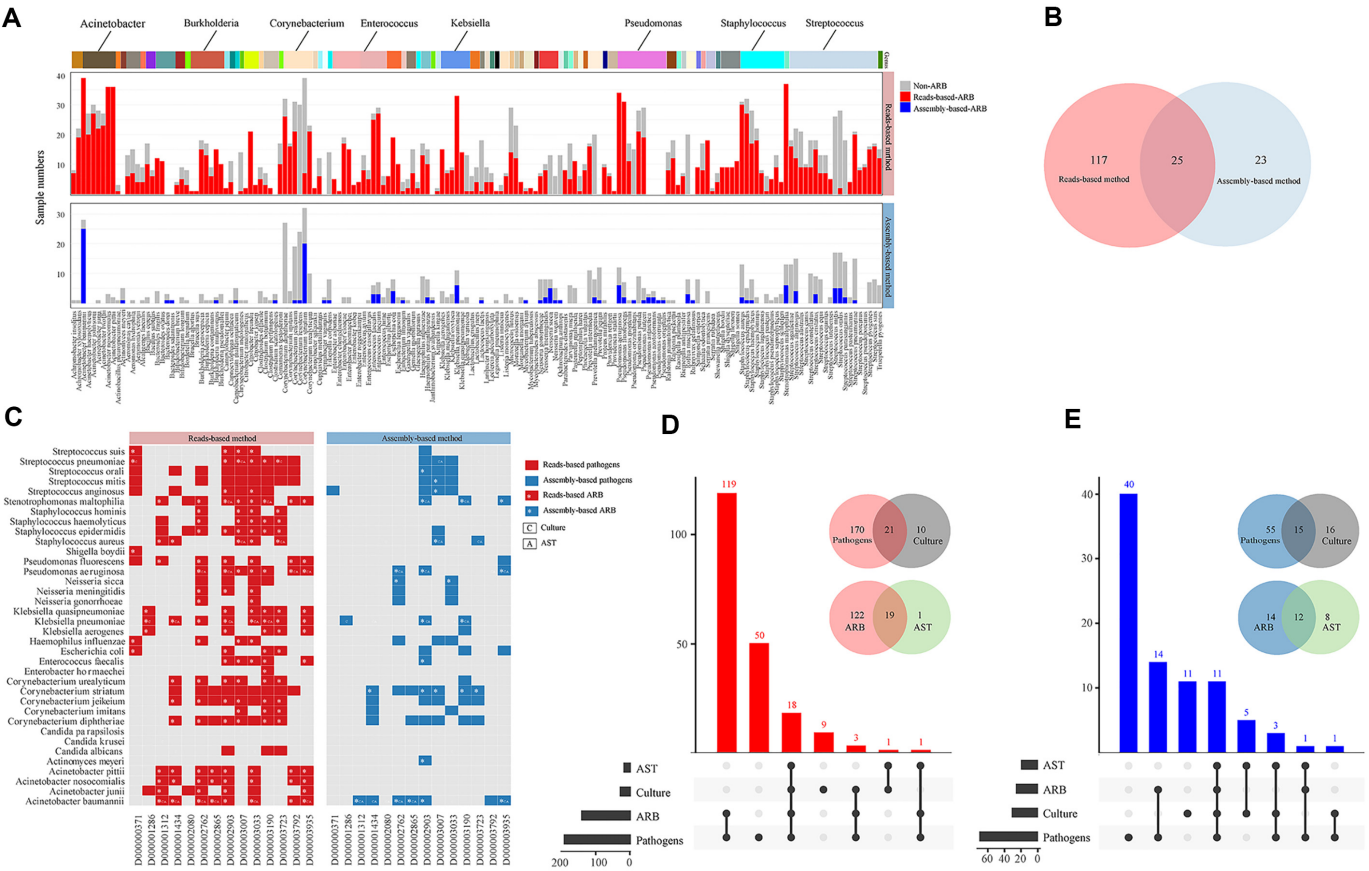
The positivity distribution of mNGS and the conventional method in 52 patients with pulmonary infections. (**A**) ARB identified as positive in 52 clinical samples by both reads-based and assembly-based methods. The upper bar plot represents the prevalence of each core genus; different colors refer to different genera. The red bar and blue bar show the frequency of ARB by the reads-based method and assembly-based method, respectively. The gray bar plot represents the non-ARB in either of these two methods. (**B**)Venn diagram showing the number distribution of ARB by two methods. (**C**) Pathogens detected by culture, antibiotic susceptibility testing (AST) results, and reads-based and assembly-based methods and analysis are shown in the heatmap. All pathogens labeled with red (reads-based) and blue (assembly-based) are the overall mNGS detection results. The parts with * imply that ARB have been confirmed by mNGS. ^*C^: culture results; ^*A^: AST results; ^*CA^: ARB confirmed by culture and AST. Upset plot showing the number of differential pathogens identified via the reads-based method (**D**) and assembly-based method (**E**) shared by combinations of culture and AST. The number above each column represents the size of differential pathogens. The set size on the right represents the number of differential pathogens in each cohort, and the connected dots represent the common differential species across connected cohorts. The number of pathogens reported by mNGS, and AST-confirmed positive cases and suspected positive cases are shown in the Venn diagram to analyze the consistency rate.

52 out of 151 samples with positive ARB by both reads-based and assembly-based methods were used for comprehensive evaluation. A total of 165 ARB was obtained by these two methods, of which 142 ARB were detected by the reads-based method (red bar), much more than the 48 detected by the assembly-based method (blue bar) (Fig. 2A). Among them, 25 ARB were obtained by both methods (Fig. 2B, Table S4). The reads-based method detected much more ARB than the assembly-based method (142 > 48). However, many congeneric species in the same genus, such as *Acinetobacter baumannii*, *Acinetobacter pittii* and *Acinetobacter nosocomialis*, were detected as ARB by the reads-based method, and only *A. baumannii* in Acinetobacter was detected as ARB by the assembly-based method. This may be caused by mapping reads directly to large data sets can inflate false-positive predictions, as reads derived from protein-coding sequences may spuriously align to other genes as a result of local sequence homology. In addition, it was found that substantial non-ARB (**gray bar**) evaluated by the assembly-based method were classified as ARB by the reads-based method. This may be caused by the reference antibiotic resistance database without the relationship between bacteria and their corresponding ARGs (ARG-bacteria attribution). The assembly-based method has the advantages of facilitating the accurate judgment of ARG-bacteria attribution, namely, ARGs identified in contiguous fragments (contigs), which were considered to belong to the bacteria. Based on the above analysis, we further compared the mNGS analysis and the culture test results.

We assessed the detection efficiency of the mNGS method and the conventional method. The reads-based method (75.5 %, 114/151) and the assembly-based method (34.4%, 52/151) yielded a higher positive detection efficiency for pathogens than the culture method (9.3%, 14/151) (Table S5). Among the 14 culture-positive samples, the reads-based method (87%, 36/42 vs. 14%, 6/42) and the assembly-based method (81%, 25/31 vs. 19%, 6/31) also showed a higher positive detection efficiency for pathogens than the culture method (Fig. 2C, culture, pathogens). ARB were detected by the reads-based method in 12 patients, 85.7% matched the culture and AST, and 64.2% (9/14) matched by the assembly-based method (Fig. 2C, ^*CA^). In total, mNGS yields a higher positive detection efficiency for pathogens and ARB compared with the conventional method.

We further assessed the consistency analysis according to the number of times pathogens in each sample. Compared with the AST results, the assembly-based method indicated consistent results with a matching rate of 46% (12/26; 12: Fig. 2C, blue cell with '*' and 'CA', 26: Fig. 2C, blue cell with '*'), which was much higher than that of the reads-based method (13%, 19/141; 19: Fig. 2C, red cell with '*' and 'CA'; 141: Fig. 2C, red cell with '*'). Fig. 2D, 2E showed the statistical results of the number of times pathogens and ARB based the two methods. These results revealed that compared to conventional detection methods, the false-positive detection rate of ARB was significantly higher by the reads-based method. Consistent with our original hypothesis, the assembly method could assist in determining the detected pathogens by the reads-based method as true ARB and improve the predictive capabilities. Intrigued by our findings, we further performed ARG-ARB network analysis to explore the relationship between the bacteria and their corresponding ARGs.

### ARG-ARB Network Analysis by the Reads-Based Method and Assembly-Based Method

**Fig. 3:**
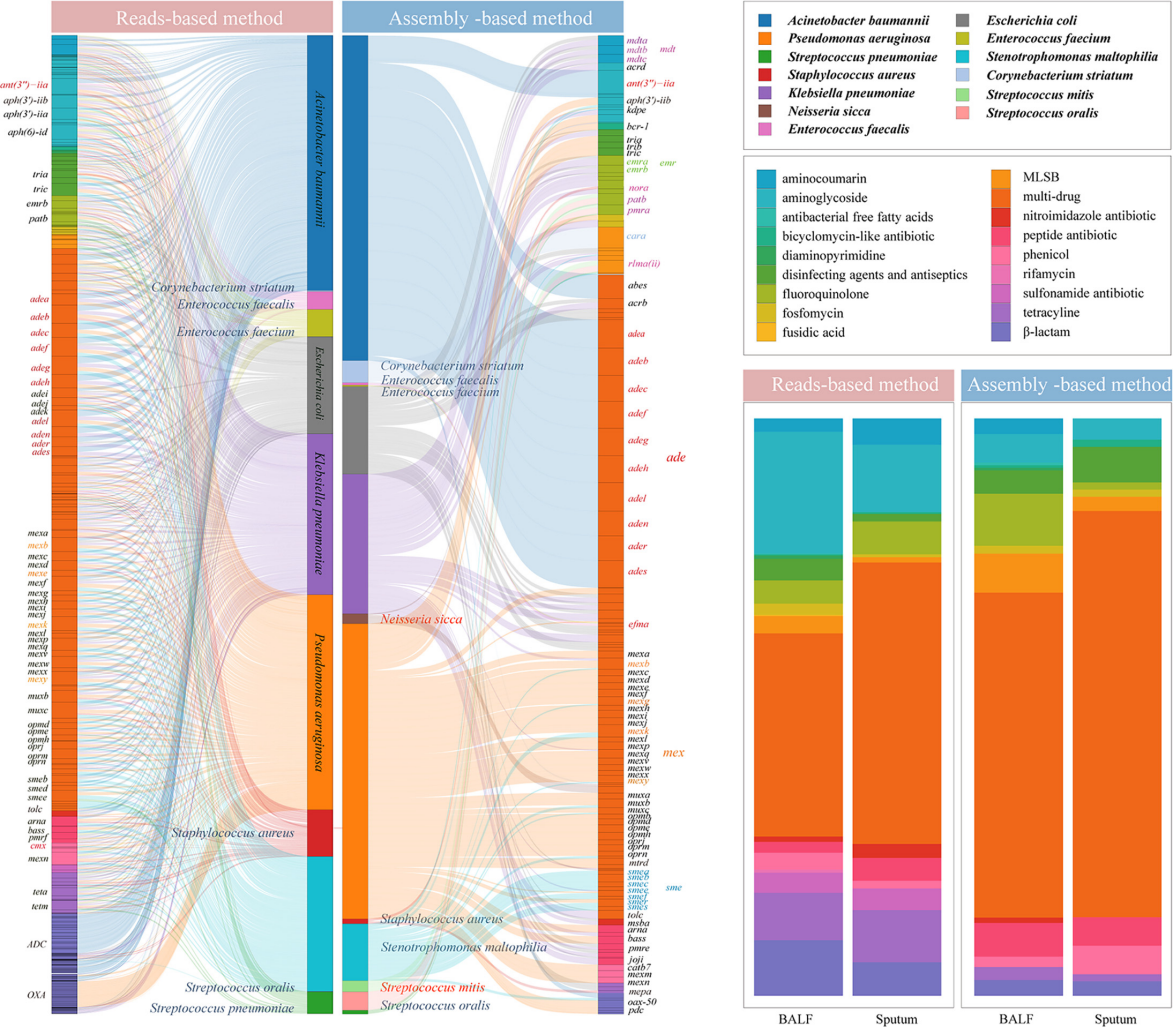
ARG-ARB network analysis. The left Sankey diagram shows the connectivity between the most prevalent ARB and their ARGs as well as the corresponding drug class. Columns on the left represent ARB and corresponding ARGs by the reads-based method, and the right represents the assembly-based method. Different colors refer to different species and different drug resistance classes. The bottom right cumulative bar graph represents the distribution of the most abundant drug resistance classes in the BALF and sputum groups based on the two methods.

To gain insight into the potential interplay of false positive predictions, we performed an ARG-ARB network analysis by reads-based and assembly-based methods. A total of 361 ARGs were detected, of which 141 were detected by the assembly-based method and 352 were detected by the reads-based method from 52 clinical samples. According to the antibiotic categories, these ARGs mainly comprised multidrug resistance (n =91), macrolide-lincosamide-streptogramin B (MLSB) (n =11), tetracycline (n =14), aminoglycoside (*n* = 36), β-lactam (n =145), aminocoumarin (n =3), phenicol (n =7), peptide (n =9), fluoroquinolone (*n* = 11), disinfecting agents and antiseptics (*n* = 10) (Table S6), which mostly belonged to the multidrug class and β-lactam antibiotic classes. Multidrug resistance of ARB to antibiotics was common in BALF and sputum samples in this study (Fig. 3, bottom right plot).

The Sankey diagram shows the connectivity between the most prevalent ARB and its ARGs as well as the corresponding drug class by the two methods. Specifically, we found substantial associations between an ARG and multiple bacteria, especially complex network results displayed by the reads-based method. Nevertheless, the assembly-based method results showed that an ARG was attributed to a particular bacterium (Fig. 3). For example, among the 101 ARGs existing in both methods, correlations between *ade* (*adea, adeb, adec, adef, adeg, adeh, adel, aden, ader, ades*) and multiple bacteria (*A. baumannii*, *K. pneumonia*, *P. aeruginosa* and *S. maltophilia*) were attributed by the reads-based method. However, they were only attributed to *A. baumannii* by the assembly-based method. In addition, 34 ARGs were detected only by the assembly-based method, including *C. striatum* (*carA*), *Neisseria sicca* (*farB, mtrC, mtrD*), and *Streptococcus mitis* (*patB, pmrA, RlmA* (II)) (Table S7, blue font), which may play a role in improving the reference antibiotic resistance database. The results revealed that the introduction of an assembly-based method could potentially contribute to determining the ARG-bacteria attribution and optimizing the antibiotic resistance database. In line with our hypothesis, the false-positive predictions by the reads-based method might be caused by the unclear ARG-bacteria attribution, an additive detection value for the improved determination of ARB and its ARGs was obtained by combining the assembly-based method.

### Identification of the Predominant ARB in Pulmonary Infection Samples

**Fig. 4:**
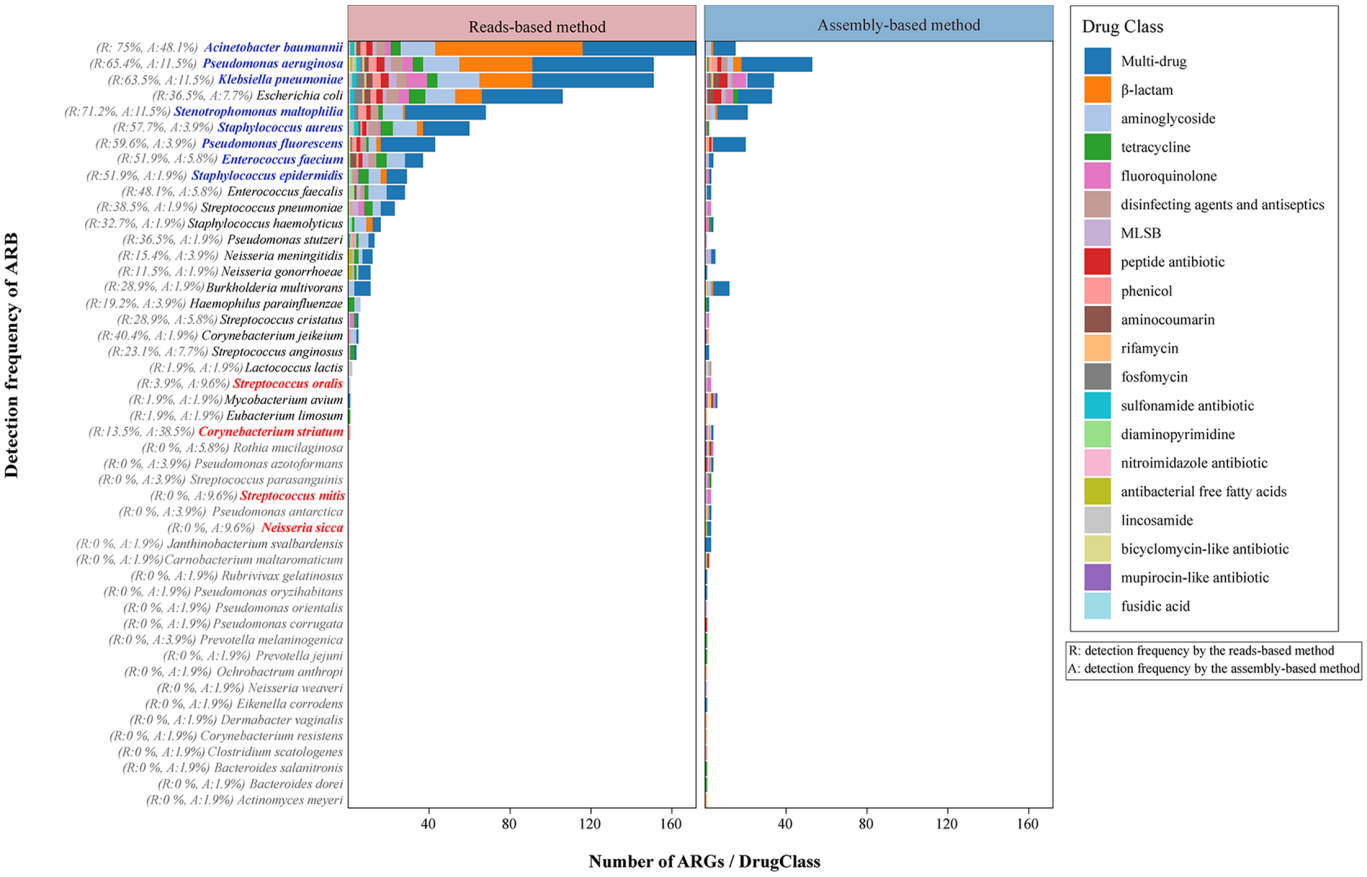
The predominant ARB and the corresponding drug class were analyzed based on the mNGS readsbased and assembly-based methods. The 48 ARB and their drug classes detected by both methods (25ARB) and only by the assembly-based method (23ARB, grey font) are shown in the bar graph. The percentages represent the detection frequency based on the 151 samples. Square colors represent the different drug class descriptions. R: detection frequency by the readsbased method; A: detection frequency by the assembly-based method.

Since the combination of the results obtained by the two methods were more effective and accurate for ARB detection, thus, we next aimed to identify the predominant ARB of the pulmonary infection patient samples. In this study, 25 ARB were identified by both methods and 23 ARB only by the assembly-based method. The ARB and its corresponding drug resistance class are also shown in Fig. 4. We chose predominant ARB according to its ranking (detection frequency). First, pathogens detected at high frequency by both methods, that is, *A. baumannii* (75.0%, 48.0%), *P. aeruginosa* (65.4%, 11.5%), *S. maltophilia* (71.2%, 11.5%), and *K. pneumonia*e (63.5%, 11.5%), were among the top four most predominant ARB (Fig. 4, blue font). Meanwhile, pathogens detected at a high frequency by the assembly-based method (Fig. 4, red font), such as *C. striatum* (38.5%), *Streptococcus oralis* (9.6%), *N. sicca* (9.6%), and *S. mitis* (9.6%), also should be paid attention.

In addition, we also explored the most prevalent ARB and its corresponding ARGs and drug classes according to CARD in this study. As follows: *A. baumannii* (*ade*, multidrug), *P. aeruginosa* (*mex*, multidrug), *K. pneumonia* (*emr*, fluoroquinolone), *S. maltophilia* (*sme*, multidrug) and *C. striatum* (*carA*, MLSB) (Table S8, red/blue font). In *A. baumannii*, *P. aeruginosa* and *S. maltophilia*, most ARGs were resistance-nodulation-division (RND)-type efflux pump genes, which conferred resistance to multiple drugs. Altogether, these results suggested the 48 ARB and its drug class in pulmonary infection patient samples, across a comprehensive analysis by the reads-based and assembly-based methods.

## Discussion

Identifying and understanding antibiotic resistance are imperative for clinical practice to treat resistant infections and for public health efforts to limit the spread of resistance. The detection of antibiotic resistance by traditional methods is limited due to complexity, time consumption and low sensitivity[19]. As shown in this study, mNGS analysis, including reads-based and assembly-based methods, effectively overcomes the deficiencies of traditional detection methods to detect antibiotic resistance. However, the ARB detected by the reads-based method showed higher false-positive predictions than that detected by the assembly-based method (Fig. 2). Additionally, the reads-based method relies more on comprehensive reference databases containing all variants of the reference genes with ARG-bacteria attribution. For example, in our study, some positive ARB, especially 23 ARB (such as *Neisseria sicca*), evaluated by the reads-based method were mistakenly classified as non-ARB due to the absence of ARG-bacteria attribution in the reference database. However, the assembly-based method, due to the contigs sequences assembling more sequences to support the ARG results [20], potentially assists the reads-based method in judging whether the detection results are valid. As the results show, the ARB detection consistency by the assembly-based method was relatively higher than that by the reads-based method (46% >13%). Taken together, these findings suggest that the assembly-based method could assist the reads-based method in determining the true ARB in clinical samples, and combining the two methods will improve the antibiotic resistance diagnostic results.

Previous studies showed that the reads-based method of mapping reads directly to large datasets can inflate false-positive predictions, as reads derived from protein-coding sequences may spuriously align to other genes as a result of local sequence homology [21]. In our study, through ARG-ARB network analysis (Fig. 3), interestingly, the introduction of an assembly-based method could contribute to determining the ARG-bacteria attribution and can effectively decrease false-positive predictions. Moreover, there were 34 ARGs undetected by the reads-based method due to the absence of ARG and ARB relationships in the reference database; examples included *C. striatum* (*carA*) and *N. sicca* (*mtrD*). These ARGs were assembled and should be attributed to the corresponding ARB, which is meaningful for complementing the antibiotic resistance database and clinical antibiotic treatment. Future studies about collecting more effective samples for higher throughput sequencing and the paired-end read information to determine the host species for the ARGs will should be considered to better present the data. As detection methods move to mNGS techniques, our knowledge about which type of bacteria carry which resistance gene(s) will become more important to ensure that the whole spectrum of bacteria is included in future surveillance studies. Antibiotic resistance database updates should be a continuous effort, as all downstream analyses depend on the accuracy of reference databases[5].

An exponential increase in antibiotic drug-resistant microbes, especially multidrug-resistant (MDR) pathogens, is a serious challenge that encompasses antibiotic therapy[22]. In this study, *A. baumannii*, *P. aeruginosa*, and *K. pneumonia*e were the predominant MDR bacteria in the pulmonary infection samples (Fig. 4, Table S5). The results are mostly consistent with previous reports that MDR pathogens, including *E. faecium*, *S. aureus*, *K. pneumonia*, *P. aeruginosa*, *A. baumannii*, and *Enterobacter* species, have been classified as ESKAPE and are the most common microbial causes of acquired infection, including pulmonary infection, and antibiotic resistance patterns are highly endemic [22, 23]. Thus, our ARB analysis highlighted the need to combine diagnostic results from reads-based and assembly-based methods for maximized predictive value. Moreover, 23 ARB detected by the assembly-based method, including *C. striatum*, *Neisseria* (*N. sicca*, *N. meningitidis*), *Streptococcus* (*S. mitis*, *S. oralis*, *S. anginosus*), *etc.*, should attract attention. In particular, emerging evidence indicates that *C. striatum*-infected individuals are at increased risk for serious infections in immunocompetent hosts and has been reported as an emerging multidrug-resistant nosocomial pathogen in recent studies [24, 25]. The reads-based method only detected the *cmx* gene (phenicol resistance) in *C. striatum* and has been reported in several studies [26, 27], which failed to detect *carA* and *mtrA* of *C. striatum* due to the incomplete antibiotic resistance database. Therefore, our study innovatively introduced the mNGS assembly-based method combined with the reads-based method, which may represent a more effective strategy for the rapid and precise detection of ARB and ARGs in clinical samples.

In addition, we also found that there was a strong relationship between ARG detection efficiency and effective reads in different types of samples (Fig. S1A). The ARG positive rate detected by the reads-based method was much higher than the assembly-based method (75.5% > 34.4%, *P* < 0.05, Fig. S1B). In the BALF and sputum samples, when the effective reads were approximately 10^7^ counts, the positive rate was 100% for both methods. The ARG-positive rate was higher with the reads-based method than with the assembly-based method, with approximately 10^6^ counts (BALF: 95.5% > 49.3%; sputum: 100% > 50%) and approximately 10^5^ counts (BALF: 71.4% > 7.1%; sputum: 66.7% > 33.3%), respectively (Fig. S1C). Herein, effective reads with more than 10^7^ counts may be more suitable for both methods to detect ARGs in BALF and sputum samples. However, more samples, including different types, are needed to verify the recommended effective read range for ARG validity analysis in future studies. Additionally, the ARG detection efficiency is also greatly affected by the filtering threshold (unique reads) [28]. When unique reads = 1, the ARG-positive rates of BALF and sputum samples were 77.6% and 58.8%, respectively. When unique reads = 8, it fell to 57.1% and 50.4%, respectively (Fig. S1D). Regarding effective reads, when unique reads = 1, the positive rates were 100%, 90%, 70% and 0% for the effective reads of 10^7^, 10^6^, 10^5^ and 10^4^ counts, respectively (Fig. S1E). Henceforth, unique reads = 1 might be introduced as the minimum filtering condition for ARG detection in the case of insufficient effective reads (≤ 10^7^). Future research should pay more attention to exploring the threshold setting for ARG verification.

There were several limitations in this study. First, the traditional pathogen detection technology is mainly based on microbial culture which depends on the vitality of pathogens and media type, incubation temperature, oxygen levels, etc. The retrospective study design did not allow for the use of PCR, complement-fixation testing, or immunofluorescence to confirm all the mNGS results. Second, due to limitations of sample volume and deep sequencing, we still need higher throughput sequencing data and comprehensive methods to detect the ARGs and assess the ARGs mapped to a pathogen are indeed hosted by that pathogen. In the future research, collecting full sample information, conduction of AST and PCR experiments, detecting species with various programs, and adjusting the threshold to further develop this research and verification. Nevertheless, our study still provided a new perspective on the applicability of mNGS in antibiotic resistance detection.

## Conclusion

The reads-based method and assembly-based method are efficient and useful diagnostic technologies for antibiotic resistance in clinical practice. Our findings demonstrated that the assembly-based method could be a supplement to the reads-based method and uncovered pulmonary infection-associated ARB and ARGs. Although many challenges remain to be overcome for its use in clinical applications, in the case of sufficient effective data, mNGS will be a revolutionary technology for clinical antibiotic resistance diagnostics, including reducing false-positive ARB predictions and promoting the antibiotic resistance database.

## Supplemental Materials

Supplementary data for this paper are available on-line only at http://jmb.or.kr.



## Figures and Tables

**Table 1:** Basic clinical characteristics of 144 patients with pulmonary infections.

Characteristics	N (%)	ARB detected (Alignment)		ARB detected (Assembly)	
		Yes	P	Yes	P
Demographics					
Age					
Mean age (years)	66.6				
<=50	27 (18.75)	25	0.172	7	0.894
>50	117(81.25)	98		44	
Sex					
Male	108 (77)	93	0.817	42	0.119
Female	36 (25)	30		9	
Antibiotic history					
Yes	119(82.63)	98	0.694	43	0.889
No	25(17.36)	25		8	
Department					
ICU	94 (65.27)	76	0.001***	28	0.001***
PD	13 (9.03)	13		10	
PCCM	23 (15.97)	21		3	
Others	14 (9.72)	13		10	
Comorbidities					
Respiratory failure	32 (22.22)	10	0.633	4	0.149
Disorders of consciousness	14 (9.72)	10		4	
Cardiovascular disease	9 (6.25)	8		5	
Malignancies	11 (7.64)	10		7	
Diabetes	6 (4.17)	5		3	
Others	17 (11.80)	15		6	
None	55 (38.19)	65		22	

ICU: intensive care unit; PD: Pneumology Department; PCCM: Pneumology Critical Care Medicine. *** *P* < 0.001

## References

[1] Iregui M, Ward S, Sherman G, Fraser VJ, Kollef MH (2002). Clinical importance of delays in the initiation of appropriate antibiotic treatment for ventilator-associated pneumonia. Chest.

[2] Ibrahim EH, Sherman G, Ward S, Fraser VJ, Kollef MH (2000). The influence of inadequate antimicrobial treatment of bloodstream infections on patient outcomes in the ICU setting. Chest.

[3] Zhang P, Chen Y, Li S, Li C, Zhang S, Zheng W (2020). Metagenomic next-generation sequencing for the clinical diagnosis and prognosis of acute respiratory distress syndrome caused by severe pneumonia: a retrospective study. PeerJ..

[4] Li N, Cai Q, Miao Q, Song Z, Fang Y, Hu B (2021). High-throughput metagenomics for identification of pathogens in the clinical settings. Small Methods.

[5] Boolchandani M, D'Souza AW, Dantas G (2019). Sequencing-based methods and resources to study antimicrobial resistance. Nat. Rev. Genet..

[6] Langmead B, Salzberg SL (2012). Fast gapped-read alignment with Bowtie 2. Nat. Methods.

[7] Li H, Durbin R (2009). Fast and accurate short read alignment with Burrows-wheeler transform. Bioinformatics.

[8] Chen H, Bai X, Gao Y, Liu W, Yao X, Wang J (2021). Profile of bacteria with ARGs among real-world samples from ICU admission patients with pulmonary infection revealed by metagenomic NGS. Infect. Drug Resist..

[9] Andreas Bremges ACM (2018). Critical assessment of metagenome interpretation enters the second round. mSystems.

[10] Carr R, Borenstein E (2014). Comparative analysis of functional metagenomic annotation and the mappability of short reads. PLoS One.

[11] Peng Y, Leung HC, Yiu SM, Chin FY (2012). IDBA-UD: a de novo assembler for single-cell and metagenomic sequencing data with highly uneven depth. Bioinformatics.

[12] Li D, Liu CM, Luo R, Sadakane K, Lam TW (2015). MEGAHIT: an ultra-fast single-node solution for large and complex metagenomics assembly via succinct de Bruijn graph. Bioinformatics.

[13] Nurk S, Meleshko D, Korobeynikov A, Pevzner PA (2017). metaSPAdes: a new versatile metagenomic assembler. Genome Res..

[14] Flicek P, Birney E (2009). Sense from sequence reads: methods for alignment and assembly. Nat. Methods.

[15] Chandrakumar I, Gauthier NPG, Nelson C, Bonsall MB, Locher K, Charles M (2022). BugSplit enables genome-resolved metagenomics through highly accurate taxonomic binning of metagenomic assemblies. Commun. Biol..

[16] Humphries R, Bobenchik AM, Hindler JA, Schuetz AN (2021). Overview of changes to the clinical and laboratory standards institute performance standards for antimicrobial susceptibility testing, M100, 31st edition. J. Clin. Microbiol..

[17] Liang Y, Dong T, Li M, Zhang P, Wei X, Chen H (2022). Clinical diagnosis and etiology of patients with *Chlamydia psittaci* pneumonia based on metagenomic next-generation sequencing. Front. Cell Infect. Microbiol..

[18] Wood DE, Salzberg SL (2014). Kraken: ultrafast metagenomic sequence classification using exact alignments. Genome Biol..

[19] Yuan J, Li W, Qiu E, Han S, Li Z (2021). Metagenomic NGS optimizes the use of antibiotics in appendicitis patients: bacterial culture is not suitable as the only guidance. Am. J. Transl. Res..

[20] Jay S Ghurye VC-E, Mihai Pop (2016). Metagenomic assembly: Overview, challenges and applications. Yale J. Biol. Med..

[21] Han D, Li R, Shi J, Tan P, Zhang R, Li J (2020). Liquid biopsy for infectious diseases: a focus on microbial cell-free DNA sequencing. Theranostics.

[22] Moore LS, Freeman R, Gilchrist MJ, Gharbi M, Brannigan ET, Donaldson H (2014). Homogeneity of antimicrobial policy, yet heterogeneity of antimicrobial resistance: antimicrobial non-susceptibility among 108,717 clinical isolates from primary, secondary and tertiary care patients in London. J. Antimicrob. Chemother..

[23] Boucher HW, Talbot GH, Bradley JS, Edwards JE, Gilbert D, Rice LB (2009). Bad bugs, no drugs: no ESKAPE! An update from the Infectious Diseases Society of America. Clin. Infect. Dis..

[24] Wang X, Zhou H, Chen D, Du P, Lan R, Qiu X (2019). Whole-genome sequencing reveals a prolonged and persistent intrahospital transmission of *Corynebacterium striatum*, an emerging multidrug-resistant pathogen. J. Clin. Microbiol..

[25] Asgin N, Otlu B (2020). Antimicrobial resistance and molecular epidemiology of *Corynebacterium striatum* isolated in a tertiary hospital in Turkey. Pathogens.

[26] Shariff M, Aditi A, Beri K (2018). *Corynebacterium striatum*: an emerging respiratory pathogen. J. Infect. Dev. Ctries.

[27] Ramos JN, Souza C, Faria YV, da Silva EC, Veras JFC, Baio PVP (2019). Bloodstream and catheter-related infections due to different clones of multidrug-resistant and biofilm producer *Corynebacterium striatum*. BMC Infect. Dis..

[28] Hunt M, Mather AE, Sánchez-Busó L, Page AJ, Parkhill J, Keane JA (2017). ARIBA: rapid antimicrobial resistance genotyping directly from sequencing reads. Microb. Genom..

